# Adherence to 24-hour movement guidelines and its association with the risk of overweight and obesity in Chinese adolescents: 2007 to 2022

**DOI:** 10.1186/s12889-026-26887-3

**Published:** 2026-03-11

**Authors:** Yi Lin, Zeng-Bao Hu, Stuart McDonald, Ke-Qin Ding, Guo-Lin Bian, Qing-Hai Gong

**Affiliations:** 1 Faculty of Humanities and Social Sciences, University of Nottingham Ningbo China, Ningbo, Zhejiang Province China; 2 Department of school health, Ningbo Municipal Center for Disease Control and Prevention, Ningbo, Zhejiang Province China

**Keywords:** Overweight, Obesity, Physical activity, Screen time, Sleep duration, 24-hour movement guidelines, Adolescents

## Abstract

**Background:**

The 24-h movement guidelines (24HGs) were developed to promote healthy lifestyles to improve the overall health status in children and youth. The aims of this study were to evaluate the prevalence of adherence to the 24HGs among Chinese adolescents from 2007 to 2022 and to investigate the associations of both overall adherence and the combinations of guideline components with the risk of overweight (OW) and obesity (OB).

**Methods:**

A multistage sampling procedure was used to draw samples aged 12 to 19 years participating in each survey wave (2007, 2012, 2017, 2022). Repeated cross-sectional data on lifestyles and anthropometry were collected through self-administered questionnaires. Participants with ≥ 60 min/day of moderate-to-vigorous physical activity (MVPA), < 2 h/day of leisure screen time (ST), and 8–11 h/day of sleep duration (SLD) were classified as adhering to all the 24HGs. Body weight status was classified based on Chinese guidelines.

**Results:**

The sample sizes for each survey wave were 861, 853, 1465 and 2599. Adherence to all the 24HGs and MVPA recommendations was consistently low across survey waves. Between 2007 and 2022, the prevalence of meeting all the 24HGs decreased from 7.20% to 3.77%, whereas the prevalence of meeting none increased from 8.94% to 11.58%. Compared to adolescents who met all the 24HGs, those meeting only one component of the 24HGs (AOR = 1.62, 95% CI: 1.05, 2.51), or ST (AOR = 1.68, 95% CI: 1.08, 2.62), or MVPA + SLD (AOR = 2.41, 95% CI: 1.30, 4.46) or none of the 24HGs (AOR = 1.87, 95% CI: 1.15, 3.04) had significantly higher risks of OW and OB.

**Conclusion:**

Across all survey waves, a small proportion of Chinese adolescents met all the 24HGs. Adolescents meeting all the 24HGs had the lowest risk of developing OW/OB among Chinese adolescents. Our findings highlight the importance of restricting leisure ST in adolescents. Future family- and school-based health education programs are needed to promote healthy lifestyles for childhood OB prevention.

**Supplementary Information:**

The online version contains supplementary material available at 10.1186/s12889-026-26887-3.

## Introduction

With the prevalence of overweight (OW) and obesity (OB) rising continuously among children and adolescents worldwide over the past five decades, OW and OB in children and adolescents have become global public health concerns. The short-term effects of childhood OW and OB are associated with physical and psychological comorbidities among children and adolescents [1], and the long-term effects of childhood OW and OB increase the likelihood of OB, chronic diseases, premature death and disability in adulthood [2, 3]. Following the global trend, the prevalence of OW among Chinese children and adolescents increased from 1% in 1985 to 13.8% in 2019, and OB increased from 0.1% in 1985 to 9.6% in 2019 [4]. Based on the report from the World Obesity Federation, more than one million children and adolescents have been identified with OB in 2030 worldwide, among which China is ranked as having the largest number of children and adolescents with OB in the world [2].

Lifestyle behaviors play a key role in the prevention and management of OB and chronic noncommunicable diseases among children and adolescents. Healthy lifestyle behaviors are closely associated with lower risks of OW and OB in adolescents [5]. Given the rapid economic and technological development over the past five decades in China, the traditional lifestyles have shifted towards ‘modern’ lifestyles, which encompass an increased incidence of risk factors that are linked to OW and OB onset, such as a sedentary lifestyle, physical inactivity, poor sleep quality, and increased use of the electronic devices. Reports from previous Chinese epidemiological studies showed that 77.3% of students aged 9–18 years engaged in less than 60 min of moderate-to-vigorous physical activity (MVPA) per day, 85.8% of students aged 6–17 years engaged in leisure screen time (ST) longer than 2 h/day, and 57.2% of students aged 8–16 years had a less than recommended sleep duration (SLD) [6–9].

Evidence shows that adequate PA, low leisure ST and sufficient SLD independently lower the risks of OW and OB and help maintain healthy body weight [10–12]. In response, the Canadian 24-Hour Movement Guidelines (24HGs) for Children and Youth were established to recommend engaging in high levels of PA, limiting sedentary behavior time and promoting sleep quality within a 24-hour period [13]. A great number of recent studies showed the connection between adherence to the 24HGs and body weight status in adolescents [14–17]. One recent systematic review and meta-analysis investigated the relationship between the 24HGs and body weight status [18]. Eight out of 15 articles concluded that children and adolescents who met all the 24HGs were more likely to have lower risks of OW and OB [18]. Previous studies reported that meeting all the 24HGs among adolescents was cross-sectionally linked to better weight status and lower risks of developing OW and OB in China [15, 19, 20], the USA [14], and Canada [21].

However, there have been few studies isolating the impact of partial adherence to the 24HGs, particularly longitudinal studies. A recent longitudinal study reported that children at 8–10 years meeting none of the 24HGs had a 1.66 SD higher BMI z-score in early adolescence (10–12 years), followed by those meeting one guideline of the 24HGs (0.95 SD), then meeting two guidelines of the 24HGs (0.53 SD) [22], indicating the need to isolate how each of the components of the 24HG contribute to the risk of OW and OB.

To date, few studies have assessed the relationship between long-term adherence to 24HGs and body weight status in China. Utilizing on repeated survey data from 2007 to 2022 in Ningbo, Zhejiang Province, the purposes of this study were: (1) to evaluate the proportion of Chinese adolescents aged 12–19 years who adhered to either the number or the combined components of the 24HGs in Ningbo from 2007 to 2022; (2) to investigate the associations of both overall and joint adherence to combinations of the 24HGs with the risk of OW and OB.

## Methods

### Study design and study population

**Fig. 1:**
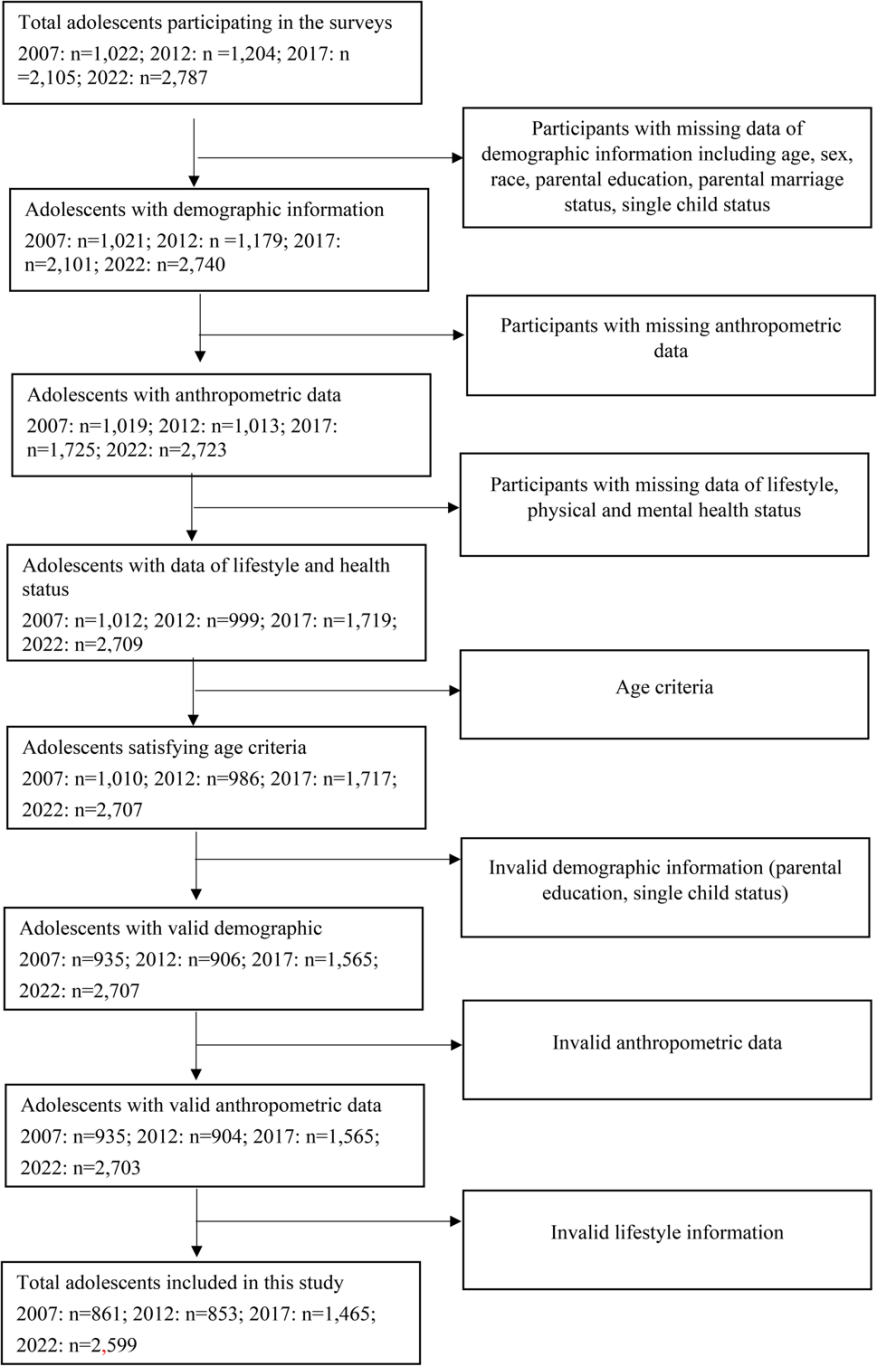
Flowchart of study population participating in Youth Risk Behavior Surveys in Ningbo from 2007 to 2022

Data were drawn from the Ningbo Youth Risk Behavior Survey (NYRBS), a large-scale, school-based cross-sectional survey, which was conducted among adolescents every 5 years starting in 2007 and ending in 2022. A multistage, stratified cluster sampling procedure was used to select a representative sample, covering socio-economic status (SES), demographics, and physical and mental health status. In stage 1, three out of ten districts were randomly selected to be representative of urban, urban-rural junction, and rural areas, respectively. In stage 2, all target schools were randomly selected from the selected districts stratified by school levels including junior middle schools, senior middle schools, and academic and vocational high schools (2007, 2012 and 2017: 3:1:1 and 2022: 12:6:6). In stage 3, two classrooms were randomly selected in each school. In stage 4, 1,022, 1,204, 2,105 and 2,751 students from the selected classes participated in surveys in 2007, 2012, 2017 and 2022, respectively (Fig. 1). Details of the study design were reported previously [23, 24].

The inclusion criteria for adolescents for this present study were: (1) children who were born in Ningbo or lived in Ningbo for at least one year; (2) children aged 12–19 years; (3) ability to understand the questions in the questionnaire and complete the questionnaire independently; (4) a permission of the class teacher; (5) the written informed consent of the parent or legal guardian for their children’s participation in the study. Adolescents were excluded if they were unable to complete the questionnaire independently, due to a physical or medical condition.

### Procedure

This study was conducted according to the Declaration of Helsinki. The study protocol and ethics were approved by Ningbo Center for Disease Control and Prevention (CDC) (No. 202201). Informed consent was obtained from all the participants. For those under 18 years of age, who are considered minors under Chinese law, additional informed consent was obtained from their parents or legal guardians, in accordance with national Guidelines for the Construction of Ethical Review Committees for Clinical Research Involving Human Beings (2023 edition) [25]. Although participants aged 18–19 years are legally competent to provide informed consent independently under Chinese law, this study was conducted in collaboration with high schools that requested parental notification for all enrolled students. Consequently, parents or legal guardians were asked to provide written acknowledgment of the study, however, their approval was not required for the students’ participation.

The questionnaires used in this study were developed based on the US 1991–2015 Youth Risk Behavior Surveillance System (YRBSS, 1991–2015) and the World Health Organization (WHO)-supported Global School-based Student Health Survey (GSHS) [26]. They covered SES, demographics, lifestyle, and physical and mental health status. The questionnaires were reviewed and approved by a panel of experts and revised following a pilot study [23, 27–29]. The finalized version has been validated and widely used in research involving Chinese school-aged students [23, 27–29].

Data were collected through self-administered anonymous paper questionnaires (henceforth referred to as questionnaires) by well-experienced researchers during the survey. All adolescents who participated in each survey were asked to complete the questionnaires independently in their classroom within 40 min under the supervision of researchers.

To ensure data quality, trained researchers implemented an on-site verification procedure to double-check all completed questionnaires. Discrepancies (e.g., in sex, weight, or height) and any missing data were identified immediately. For those items with misreported information, adolescents were asked to review and correct their answers. For missing items, adolescents were asked to complete the omitted questions again if they were willing and able to do so. This on-site quality control procedure helped in minimizing data errors and improving completeness.

### Assessment of body weight

Adolescents reported their anthropometric data based on their most recent school annual health check. Anthropometric measurements were taken with participants wearing light clothing and without shoes. Body weight was recorded to the nearest 0.1 kg using a calibrated electronic scale, and standing height was measured to the nearest 0.1 cm using a portable stadiometer. Body mass index (BMI), which identifies body weight status, was calculated as weight (kg)/height^2^ (m^2^). Body weight status of adolescents was classified into underweight (UW), normal weight (NW), OW and OB, using age-specific and sex-specific cut-off values from the National Health Commission of the People’s Republic of China (screening for OW and OB among school-aged children and adolescents, WS/T 586—2018) [30].

### 24-hour movement behaviors

The level of PA was assessed using the same question in all four waves: ‘How many days were you able to do at least 60 minutes of MVPA over the past week?’. MVPA was categorized as 7 days or fewer than 7 days, following the guidelines by both the Chinese Guidelines on Physical Activity for Children and Adolescents and the Canadian 24HGs [31, 32].

ST was assessed using the consistent question across all survey waves ‘How long did you use electronic devices (e.g. television, smartphone, computer and tablets) for leisure over the past week?’. To meet the guideline of leisure ST, the time spent on electronic devices by adolescents must be less than 2 h daily according to the Canadian 24HGs [31].

All students reported their average SLD every night for the past week. In accordance with the Canadian 24HGs, adolescents aged 12–13 years who reported 9–11 h of sleep per day and those aged 14–19 years who had 8–10 h of sleep per day were classified as meeting the sleep recommendation [31].

### Covariates

Adolescents reported data on demographics, socioeconomic status (SES), and lifestyle behaviors. Demographic variables included sex, age (< 16, ≥ 16, reflecting entry into senior high school in the Chinese education system), ethnicity (Han, others), school type (junior middle school, senior middle school/ high school), parental marriage status (nuclear family, separated/single-parent family and others) and single child status.

SES indicators comprised residence area (urban vs. urban-rural junction/rural), the highest degree of parental education, categorized according to completion of China’s nine-year compulsory education: (1) no parents completing junior middle school education; (2) one parent completing junior middle school education; and (3) both parents completing junior middle school education.

Lifestyle behaviors included alcohol consumption, sugar-sweetened beverage intake, and junk food consumption. Alcohol consumption for the past month was categorized into three levels: never, 1–10 times/month, > 10 times/month, in accordance with the WHO recommendation for risk level reduction in alcohol consumption [33]. Following the WHO guideline on the intake of free sugars for reduction in the risk of chronic diseases in children [34], sugar-sweetened beverage intake for the past week was classified as never, once/day, and ≥ 2 times/day. Junk food consumption for the past week was categorized as: never, 1–3 times/week, > 3 times/week, in alignment with the Chinese Dietary Guideline, which recommends reducing energy-dense, nutrient-poor foods (e.g., processed meats, fried snacks) to promote healthy dietary patterns [35].

### Statistical analysis

Descriptive statistics were reported using number and percentages for category variables and mean and standard deviation (SD) for continuous variables to describe adolescents’ characteristics, the prevalence of body weight status, the proportion of adolescents adhering to all the 24HGs and mean values of anthropometry for each wave. Statistical differences in the prevalence and mean values across waves were examined using the Cochran Armitage test and ANOVA, respectively.

A binary generalized linear model (GLM) was used to examine associations between adherence to the 24HGs and OW and OB via three models: (1) Model 1: crude model; (2) Model 2: adjusted for demographic and SES; (3) Model 3: further adjusting for lifestyle behaviors and wave. Adherence to all the 24HGs was the reference category in the models. Multicollinearity among covariates was evaluated by variance inflation factors (VIF).

Results were considered statistically significant at a two-tailed level of 0.05. Statistical analysis was conducted using the STATA statistical software package version 18 (2021).

## Results

In total, 861, 853, 1,465 and 2,599 adolescents were included from each survey wave in the present study. The general characteristics of total participants from 2007 to 2022 are presented in Table 1. A total of 5,778 adolescents with mean age of 15.92 years participated in all 4 surveys, of whom 48.62% were boys. The majority of adolescents in each survey wave resided in urban-rural junction/rural areas, came from nuclear families, lived with family members, and reported no consumption of sugar-sweetened beverages, junk food or alcohol.

**Table 1:** Adolescents’ characteristics in Youth Risk Behavior Survey from 2007 to 2012

Variables	Category/Items	2007 (*n* = 861)	2012 (*n* = 853)	2017 (*n* = 1,465)	2022 (*n* = 2,599)	*P* for trend*
		*n* (%)				
Sex						0.619
	Male	477 (55.40)	357 (41.85)	673 (45.94)	1,302 (50.10)	
	Female	384 (44.60)	496 (58.15)	792 (54.06)	1,297 (49.90)	
Age (years)						< 0.001
	12.0-15.9	408 (47.30)	308 (36.11)	775 (52.90)	1,328 (51.10)	
	16.0-19.9	453 (52.61)	545 (63.89)	690 (47.10)	1,271 (48.90)	
School type						0.001
	Junior	454 (52.73)	439 (51.47)	688 (46.96)	1,224 (47.10)	
	Senior	407 (47.27)	414 (48.53)	777 (53.04)	1,375 (52.90)	
Area of residence						< 0.001
	Urban	185 (21.49)	262 (30.72)	544 (37.13)	899 (34.59)	
	Urban-rural junction/rural	676 (78.51)	591 (69.28)	921 (62.87)	1,700 (65.41)	
Ethnicity						< 0.001
	Han	854 (99.19)	840 (98.48)	1,429 (97.54)	2,505 (96.38)	
	Others	7 (0.81)	13 (1.52)	36 (2.46)	94 (3.62)	
Single child status						< 0.001
	Yes	571 (66.32)	565 (66.24)	943 (64.37)	1,209 (46.52)	
	No	290 (33.68)	288 (33.76)	522 (35.63)	1,390 (53.48)	
Parental education						0.846
	No parents completing junior middle school	485 (56.33)	432 (50.64)	712 (48.60)	1,326 (51.02)	
	One parent completing junior middle school	193 (22.42)	191 (22.39)	499 (34.06)	836 (32.17)	
	Both parents completing junior middle school	183 (21.25)	230 (26.96)	254 (17.34)	437 (16.81)	
Parental marriage						< 0.001
	Nuclear family	805 (93.50)	781 (91.56)	1,341 (91.54)	2,307 (88.76)	
	Single-parent family	42 (4.88)	69 (8.09)	124 (8.46)	292 (11.24)	
	Others	14 (1.63)	3 (0.35)	NA	NA	
Living situation						< 0.001
	Dorm	347 (40.30)	354 (41.50)	546 (37.27)	1,019 (39.21)	
	With family	470 (54.59)	450 (52.75)	870 (59.39)	1,484 (57.10)	
	Apart from family	44 (5.11)	49 (5.74)	49 (3.34)	96 (3.69)	
Sugar-sweetened beverages consumption						< 0.001
	Never	499 (57.96)	713 (83.59)	1,221 (83.34)	2,331 (89.69)	
	1 time/day	163 (18.93)	69 (8.09)	111 (7.58)	146 (5.62)	
	≥ 2 times/day	199 (23.11)	71 (8.32)	133 (9.08)	122 (4.69)	
Junk food consumption						0.001
	Never	570 (66.20)	532 (62.37)	803 (54.81)	1,547 (59.52)	
	1–3 times/week	274 (31.82)	307 (35.99)	626 (42.73)	1,012 (38.94)	
	> 3 times/week	17 (1.97)	14 (1.64)	36 (2.46)	40 (1.54)	
Alcohol consumption						0.001
	Never	626 (72.71)	617 (72.33)	1,159 (79.11)	2,251 (86.61)	
	1–10 times/month	226 (26.25)	226 (26.49)	292 (19.93)	330 (12.70)	
	> 10 times/month	9 (1.05)	10 (1.17)	14 (0.96)	18 (0.69)	
Weight status						< 0.001
	UW/NW	797 (92.57)	802 (94.02)	1,323 (90.31)	2,236 (86.03)	
	OW/OB	64 (7.43)	51 (5.98)	142 (9.69)	363 (13.97)	
24-hour movement behaviors						
	None	77 (8.94)	75 (8.79)	137 (9.35)	301 (11.58)	0.006
	One	784 (91.06)	778 (91.21)	1,328 (90.65)	2,298 (88.42)	0.006
	Two	435 (50.52)	391 (45.84)	660 (45.05)	911 (35.05)	< 0.001
	All	62 (7.20)	40 (4.69)	99 (6.76)	98 (3.77)	< 0.001
		Mean (SD)				
Anthropometry						
	Weight (kg)	52.49 (10.71)	52.30 (9.09)	54.49 (10.56)	56.93 (12.40)	< 0.001
	Height (cm)	164.25 (8.71)	164.55 (8.32)	166.36 (8.50)	166.83 (8.46)	< 0.001
	BMI (kg/m^2^)	19.33 (2.91)	19.26 (2.70)	19.61 (3.06)	20.36 (3.64)	< 0.001

*AOR* Adjusted odds ratio, *CI* Confidence interval, *MVPA* Moderate-to-vigorous physical activity, *ST* Screen time, *SLD* Sleep duration, *UOR* Unadjusted odds ratio

* The Cochran-Armitage test for category variables and one-way ANOVA for continuous variables were used to examine time trend across four waves

The trend in the prevalence of adolescents with OW and OB (7.43%) in 2007 significantly increased to 13.97% in 2022 along with an increase in BMI from 19.33 to 20.36 kg/m^2^ (Table 1). The proportion of adolescents meeting all the 24HGs significantly declined from 7.20% in 2007 to 3.77% in 2022, and an increasing trend in the prevalence of meeting none of the 24HGs to 11.58% in 2022 from 8.94% in 2007.

**Fig. 2:**
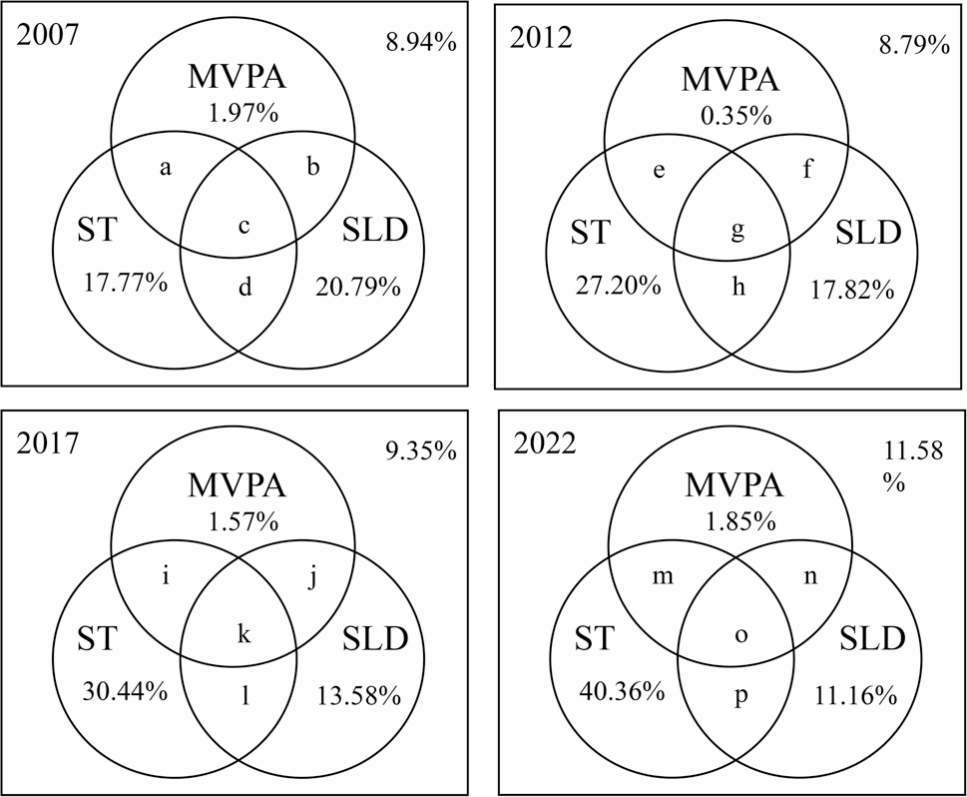
Proportions of Chinese adolescents meeting 24-Hour Movement Guidelines in 2007, 20012, 2017, and 2022. MVPA: moderate-to-vigorous physical activity; ST: screen time; SLD: sleep duration. Each circle represents one component behavior of the 24-Hour Movement Guidelines. Percentages in each circle represent the proportion of adolescents meeting one component behavior of the 24-Hour Movement Guidelines, while percentages in the overlapping of areas show the proportion of adolescents meeting multiple components behaviors simultaneously. a: 4.88%; b: 3.48%; c: 7.20%; d: 34.96%; e: 2.58%; f: 2.46%; g: 4.69%; h: 36.11%; i: 5.87%; j: 2.87%; k: 6.76%; l: 29.56%; m: 3.85%; n: 1.54%; o: 3.77%; p: 25.89%

In addition, the trends in adherence to the combinations of the 24HGs components were significant across waves. The proportion of adolescents meeting MVPA was very low in all waves (Fig. 2). Notably, there were decreasing trends in the prevalence of only meeting SLD, MVPA + SLD, and ST + SLD from 20.79%, 3.48% and 34.96% in 2007 to 11.16%, 1.54% and 25.89% in 2022, respectively, while the prevalence of only meeting ST increased from 17.77% to 40.36% across 15 years.

Table 2 demonstrates the associations between the number or the combinations of the 24HGs components met, and the risk of OW and OB among adolescents. All 5,778 adolescents were included collectively in the regression. The results reveal that meeting none of the 24HGs (UOR = 1.72, 95% CI: 1.07, 2.76, *P* = 0.025), only meeting MVPA (UOR = 2.34, 95% CI: 1.19, 4.60, *P* = 0.014) and meeting MVPA + SLD (UOR = 2.41, 95% CI: 1.32, 4.41, *P* = 0.004) were associated with a higher risk of OW and OB, compared to meeting all the 24HGs, in Model (1). After controlling for demographics and SES, meeting one component, only meeting ST, only meeting MVPA, meeting MVPA + SLD, and meeting none of the 24HGs were positively significant in subsequent Model (2). Further controlling for additional lifestyle behaviors and wave, meeting one component (AOR = 1.62, 95% CI: 1.05, 2.51, *P* = 0.029), only meeting ST (AOR = 1.68, 95% CI: 1.08, 2.62, *P* = 0.022), meeting MVPA + SLD (AOR = 2.41, 95% CI: 1.30, 4.46, *P* = 0.005), and meeting none of the 24HGs (AOR = 1.87, 95% CI: 1.15, 3.04, *P* = 0.011) were associated with higher odds of OW and OB in Model 3.

**Table 2:** Associations between the number and the combinations of 24-Hour Movement Guidelines met and the risk of overweight and obesity in adolescents

	Model 1^a^			Model 2^a^			Model 3^a^		
	UOR	95% CI	*P*	AOR	95% CI	*P*	AOR	95% CI	*P*
Number of 24-Hour Movement Guidelines									
All	1			1			1		
None	1.72	1.07,2.76	0.025	2.04	1.26,3.30	0.004	1.87	1.15,3.04	0.011
One	1.42	0.93,2.17	0.107	1.76	1.14,2.71	0.01	1.62	1.05,2.51	0.029
Two	1.12	0.73,1.74	0.601	1.38	0.89, 2.14	0.154	1.34	0.86,2.09	0.195
Combination of 24-Hour Movement Guidelines									
All	1			1			1		
None	1.72	1.07, 2.76	0.025	2.04	1.26, 3.30	0.004	1.87	1.15, 3.04	0.011
MVPA	2.34	1.19, 4.60	0.014	2.02	1.01, 4.02	0.046	1.88	0.94, 3.76	0.074
SLD	1.15	0.72, 1.85	0.556	1.41	0.87, 2.27	0.163	1.44	0.89, 2.33	0.138
ST	1.50	0.97, 2.31	0.067	1.90	1.23, 2.95	0.004	1.68	1.08, 2.62	0.022
MVPA + SLD	2.41	1.32, 4.41	0.004	2.32	1.26, 4.27	0.007	2.41	1.30, 4.46	0.005
MVPA + ST	1.11	0.61, 2.01	0.729	1.07	0.59, 1.94	0.836	1.01	0.56, 1.85	0.967
ST + SLD	1.04	0.67, 1.61	0.878	1.35	0.86, 2.11	0.193	1.31	0.83, 2.06	0.244

*AOR* Adjusted odds ratio, *CI* Confidence interval, *MVPA* Moderate-to-vigorous physical activity, *ST* Screen time, *SLD* Sleep duration, *UOR* Unadjusted odds ratio

^a^Model 1: the crude model; Model 2: adjusted for age, sex, school type, ethnicity, single child status, parental education status, parental marriage status, living situation; Model 3: further adjusted for consumption of sugar-sweetened beverages, junk food, and alcohol, and survey wave; Reference group: MVPA + ST+SLP for all the models

## Discussion

This school-based study with four-repeated survey waves is the first long-term study to evaluate the proportion of adolescents adhering to the number or the combined components of the 24HGs, and to investigate these associations with OW and OB among Chinese adolescents aged 12–19 years. The prevalence of meeting all the 24HGs in each wave was low among Chinese adolescents. A decreasing trend in the prevalence of meeting all 24HGs and an increasing trend in the proportion of adolescents meeting none of the 24HGs was observed across 4 waves. Our findings indicate that meeting none of the 24HGs and meeting one component of the 24HGs were associated with an increase in the risk of OW and OB among adolescents, compared to meeting all the 24HGs. Likelihood of OW and OB was higher among adolescents, in particular, who met only ST guideline and MVPA and SLD guidelines.

The trend in BMI and the prevalence of adolescents with OW and OB increased gradually from 2007 to 2022 in our study, which was supported by a systematic review and meta-analysis [36]. In order to comprehensively understand that the 24HGs are applied to Chinese adolescents, it is necessary to assess the characteristics of the 24HGs and evaluate the prevalence of adherence to the 24HGs among Chinese adolescents. Consistent with the reports from the China Health and Nutrition Survey from 2004 to 2015, our results showed a low prevalence of adherence to all the 24HGs in each wave and a decreasing trend in the prevalence across 15 years [19]. The prevalence of Chinese adolescents meeting all the 24HGs in 2022 in our study was lower than that among Chinese children and adolescents aged 7–18 years from one municipality and six capital cities in 2019 (7.3%) [15], but was close to that on the national level in 2015 (3.4%) [19]. Compared to foreign countries, the prevalence in 2022 in our study was lower than that of American adolescents aged 15–16 years in 2021 (4.88%) [14], children aged 9–11 years on the international level in 2013 (7.2%) [17] and Canadian adolescents aged 12–17 years in 2013 (5.5%) [37].

The rapid economic growth has resulted in the transition of lifestyle and nutrition towards ‘Western’ style for the past 5 decades, which may be a key factor causing poor quality of life [38]. In particular, the prevalence of meeting the MVPA guideline was very low in our study [15]. Therefore, the low adherence rate of the MVPA guideline is an important factor resulting in the low prevalence of adherence to all the 24HGs among adolescents in our study. Another possible explanation for the low prevalence of adherence to all the 24HGs in 2022 in our study might be lockdown during the zero-Covid policy period causing changes in lifestyles towards unhealthy lifestyle patterns [39]. Moreover, over the 15-year study period, the declining trend in adherence to sleep guideline along with the increasing trend in leisure screen time guideline may reflect the growing academic demands and academic performance pressures in adolescents which could encroach on sleep time [40, 41]. Therefore, subgroup analysis stratified by pandemic period (pre- vs. post-2020) and by period of widespread smartphone adoption (pre- vs. post 2017) will be conducted in future studies.

Healthy lifestyle behaviors play an important role in the prevention of OW and OB in adolescents [5]. In China, adolescents’ lifestyles transitioned towards a lack of PA, poor sleep quality, and an increase in leisure ST on electronic devices [8, 9]. Increasing evidence reveals that physical inactivity, ubiquitous screen uses on devices, and insufficient SLD are key causes of impacts on physical and mental health, physical decline, OW and OB, and chronic diseases [11, 12, 42, 43]. The 24HGs, developed to promote healthy lifestyle behaviors in children and adolescents for improvement of health status and disease prevention, have gained interest to behavioral and public health scientists [13]. Better understanding the link of meeting the 24HGs to OB risks is of vital importance to design effective public health initiatives and health intervention programs for OB prevention. One recent systematic review concluded that meeting all 24HGs benefited a healthy body weight status and body composition in children and adolescents [44].

Our results show that adolescents who met none of the 24HGs had a higher likelihood of being OW or obese, which is in line with numerous cross-sectional studies from China and the Czech Republic among adolescents [14–16]. With limited evidence from longitudinal studies, two recent longitudinal studies following short-term follow-up indicated that meeting none of the 24HGs in children was associated with higher adiposity and cardio-metabolic risks in early adolescence (10–12 years) [22, 45]. In addition, children aged 8–10 years meeting fewer components of the 24HGs were shown to be associated with higher adiposity in late adolescence (15–17 years) compared to those meeting all 24HGs [22].

Besides adherence to all the 24HGs, lower risk of OW and OB was found to be associated with meeting more components of the 24HGs in our study, which is consistent with those reports from previous studies [14, 16]. Therefore, our findings highlight the promotion of a comprehensive lifestyle behavior strategy, rather than promoting a single behavior, for prevention of OW and OB in Chinese children and adolescents. However, meeting any of two components of the 24HGs was not associated with OW and OB in our study. A possible explanation may be very low rate of adherence to all the 24HGs, the MVPA guideline in particular.

In line with previous cross-sectional studies using the same categorization threshold [16, 46], adolescents meeting MVPA and SLD guidelines had a 2.41 times higher risk of OW and OB in our study, compared to those meeting all the 24HGs. Notably, adolescents who met both MVPA and SLD guidelines were associated with higher odds of OW and OB than adolescents who met only the ST guideline. Our findings suggest that excessive leisure ST was more strongly associated with higher odds of OW and OB during adolescence than PA and sleep alone. Reports from previous studies are in accordance with our findings, which revealed that meeting the ST guideline was associated with lower risks of OB-related indicators whether or not PA and sleep guidelines were met [16, 17]. Several cross-sectional studies indicated that only meeting PA or sleep guideline, and combinations thereof were not associated with OW and OB in adolescents [46, 47]. The potential mechanism can be that leisure ST on electronic devices contributing to prolonged sedentary time results in an inactive physical lifestyle and short SLD, which can cause a lower basal metabolic rate, endocrine and metabolic alterations, and heightened appetite [48]. Therefore, more caloric intake and decreased total energy expenditure can lead to an increase in OB-related indicators. In addition, the excessive use of electronic devices increase adolescents’ exposure to food marketing through digital platforms, which can trigger adolescents unplanned and unconscious snacking behaviors especially on high-calorie, ultra-processed foods rich in sugar and fat [49]. This dietary pattern contributes to excess energy intake and disrupt energy homeostasis, consequently increased risk of OW and OB. Moreover, overuse of electronic devices before bed time is a behavior more common among adolescents with greater bedtime autonomy among adolescents due to less parental restriction [50]. Pre-sleep screen exposure can delay sleep onset, suppress melatonin secretion, and disrupt circadian rhythms, which may dysregulate appetite-regulating hormones (e.g., leptin and ghrelin) [51–53], thereby increasing the risk of weight gain.

Our findings are consistent with a previous nationwide study of Chinese children and adolescents [54], which reported that meeting the MVPA guideline, the SLD guideline, or both MVPA and SLP guidelines were associated with higher risk of OW and OB in total sample. In sex- and age-stratified analyses, the association between meeting both MVPA and SLD guidelines, and OW and OB was statistically significant among girls in grades 4–6 [54]. The non-significant associations between MVPA and ST guidelines, and ST and SLD guidelines, and OW and OB in our study may be modified by sex and age. Biological differences related to sex and developmental stage (e.g. sex steroid hormones), which play a key role in regulating of fat distribution and fat mass accumulation [55], could influence the associations. Another potential explanation is dietary behaviors and eating patterns [56], as excessive energy intake, and energy-dense, nutrient-poor diets could contribute to OW and OB.

### Strengths and limitations

This is the first large-scaled repeated cross-sectional study to investigate the associations of adherence to either the number or the combined components of the 24HGs with OW and OB among Chinese adolescents. Our findings could provide insightful evidence to the limited literature on associations between adherence to the 24HGs, and OW and OB and development of health programs to improve movement behaviors among Chinese adolescents. However, several limitations should be noted. First, causality cannot be inferred due to the nature of the observational study design. Second, our sample was drawn from Ningbo, a relatively developed coastal city in eastern China. Consequently, our findings may not be generalizable to adolescents in rural or less-developed regions in China, particularly western China, where socioeconomic conditions, educational resources, family structures, and lifestyle environments differ substantially. Therefore, the policy recommendations derived from our study should be applied with caution and adapted to local conditions when considering geographic and socioeconomic contexts. Nevertheless, this study can provide a valuable reference and methodological framework for understanding adolescents’ 24-hour movement behaviors and their health outcomes across China. Our findings offer insights into future research to investigate potential disparities in 24-hour movement behaviors and their health impacts in diverse socioeconomic and geographic regions in China. Third, a potential point is the use of a 7 days/week cut-off threshold to identify MVPA adherence, aligns with the emphasis on daily PA in both the Chinese Guidelines on Physical Activity for Children and Adolescents and the Canadian 24HGs [31, 32]. This dichotomizing categorization may have simplified the continuous nature of PA behavior and misclassified adolescents who had PA on 3–6 days/week as non-adherent, which might have potentially influenced the estimated association with the risk of adolescent OW and OB. However, using an alternative cut off point (≤ 3 vs. >3 days/week) in a sensitivity analyses yielded similar estimates and confidence intervals, with the associations remaining statistically non-significant (Supplementary Table 1). That indicates that our main findings are robust based on using a 7 days/week cut-off threshold. Then, all data on lifestyle behaviors, demographics and SES collected through self-reported questionnaires can cause recall bias. Another limitation is that our study only focused on the duration of recreational screen time as a component of the 24-Hour Movement Behaviors. Given the rapid technological evolution over the study period, the lack of detail on usage patterns limits a comprehensive understanding of how specific screen-based behaviors may differentially impact the risk for OW and OB. Future studies, therefore, should examine these specific usage patterns and their differential effects on OW and OB. Moreover, childhood body weight, and parental and nutritional health status (e.g. weight status, cardio-metabolic indicators) were not collected in our study. These factors are known to contribute to an increased risk of adolescent OW and OB [57, 58]. Finally, self-reported weight and height relied on adolescents’ memory and psychological bias, which might affect the accuracy of actual BMI and body weight status. Therefore, it could affect the bias and accuracy of the associations. Well-designed longitudinal research is needed to verify causality of lifestyle patterns and OW/OB in Chinese children and adolescents across diverse socioeconomic regions.

## Conclusion

Our study showed a declining trend in the prevalence of meeting all 24HGs and an increasing trend in the prevalence of meeting none of the 24HGs from 2007 to 2022. The prevalence of adherence to the 24HGs among adolescents in each wave was low. Adolescents meeting all the 24HGs had the lowest risk of developing OW/OB. Among the components of the 24HGs, leisure ST showed the strongest association with OW and OB. Compared to adolescents meeting all components of the 24HGs, those meeting none of the 24HGs, only the ST guideline, or the combination of the MVPA and SLD guidelines had significantly higher odds of OW and OB. Our findings provide evidence for future longitudinal studies and health intervention program to improve policy making and school health strategies for promoting healthy lifestyle behaviors, and OW and OB prevention.

## Supplementary Information

Supplementary Material 1.

## Data Availability

The data is not publicly available due to privacy or ethical restrictions. If there is a reasonable request, it can be obtained from the corresponding authors.

## References

[1] Pulgarón ER. Childhood obesity: a review of increased risk for physical and psychological comorbidities. Clin Ther. 2013;35(1):A18–32. 23328273 10.1016/j.clinthera.2012.12.014 PMC3645868

[2] Jebeile H, Kelly AS, O’Malley G, Baur LA. Obesity in children and adolescents: epidemiology, causes, assessment, and management. Lancet Diabetes Endocrinol. 2022;10(5):351–65. 35248172 10.1016/S2213-8587(22)00047-X PMC9831747

[3] Reilly JJ, Kelly J. Long-term impact of overweight and obesity in childhood and adolescence on morbidity and premature mortality in adulthood: systematic review. Int J Obes. 2011;35(7):891–8. 10.1038/ijo.2010.222 20975725

[4] Dong YH, Chen L, Liu JY, Ma T, Zhang Y, Chen MM, Zhong PL, Shi D, Hu PJ, Li J, et al. [Epidemiology and prediction of overweight and obesity among children and adolescents aged 7–18 years in China from 1985 to 2019]. Zhonghua Yu Fang Yi Xue Za Zhi. 2023;57:11–9. 36854438 10.3760/cma.j.cn112150-20220906-00881

[5] Calcaterra V, Verduci E, Vandoni M, Rossi V, Fiore G, Massini G, Berardo C, Gatti A, Baldassarre P, Bianchi A, et al. The Effect of Healthy Lifestyle Strategies on the Management of Insulin Resistance in Children and Adolescents with Obesity: A Narrative Review. Nutrients. 2022;14:21. 10.3390/nu14214692 PMC9657567 36364954

[6] Zhang X, Song Y, Yang TB, Zhang B, Dong B, Ma J. [Analysis of current situation of physical activity and influencing factors in Chinese primary and middle school students in 2010]. Zhonghua Yu Fang Yi Xue Za Zhi. 2012;46(9):781–8. 23157880

[7] Song C, Gong W, Ding C, Yuan F, Zhang Y, Feng G, Chen Z, Liu A. Physical activity and sedentary behavior among Chinese children aged 6–17 years: a cross-sectional analysis of 2010–2012 China National Nutrition and health survey. BMC Public Health. 2019;19(1):936. 31296189 10.1186/s12889-019-7259-2 PMC6624983

[8] Zhu Z, Tang Y, Zhuang J, Liu Y, Wu X, Cai Y, Wang L, Cao ZB, Chen P. Physical activity, screen viewing time, and overweight/obesity among Chinese children and adolescents: an update from the 2017 physical activity and fitness in China-the youth study. BMC Public Health. 2019;19(1):197. 30767780 10.1186/s12889-019-6515-9 PMC6376726

[9] Chen H, Wang LJ, Xin F, Liang G, Chen Y. Associations between sleep duration, sleep quality, and weight status in Chinese children and adolescents. BMC Public Health. 2022;22(1):1136. 35668374 10.1186/s12889-022-13534-w PMC9172025

[10] Crowe M, Sampasa-Kanyinga H, Saunders TJ, Hamilton HA, Benchimol EI, Chaput JP. Combinations of physical activity and screen time recommendations and their association with overweight/obesity in adolescents. Can J Public Health. 2020;111(4):515–22. 32285346 10.17269/s41997-020-00313-6 PMC7438465

[11] Miller MA, Kruisbrink M, Wallace J, Ji C, Cappuccio FP. Sleep duration and incidence of obesity in infants, children, and adolescents: a systematic review and meta-analysis of prospective studies. Sleep. 2018;41(4)1–19. 10.1093/sleep/zsy018 29401314

[12] Moitra P, Madan J, Verma P. Independent and combined influences of physical activity, screen time, and sleep quality on adiposity indicators in Indian adolescents. BMC Public Health. 2021;21(1):2093. 34781921 10.1186/s12889-021-12183-9 PMC8591930

[13] The Government of Canada: 24 hour movement guidelines for children and youth: An integration of physical activity, sedentary behaviour, and sleep. 2016. https://www.canada.ca/en/public-health/services/health-promotion/healthy-living/physical-activity/24-hour-movement-guidelines-children-youth.html. Accessed 1 June 2025.

[14] Su Y. Compliance with the 24-hour movement guidelines and weight status: results from 40,970 adolescents. Front Public Health. 2024;12:1472188. 40123785 10.3389/fpubh.2024.1472188 PMC11925785

[15] Sun Y, Liu Y, Yin X, Li M, Zhang T, Zhang F, et al. Proportion of Chinese Children and Adolescents Meeting 24-Hour Movement Guidelines and Associations with Overweight and Obesity. Int J Environ Res Public Health. 2023;20(2):1408. 10.3390/ijerph20021408 PMC9859181 36674163

[16] Jakubec L, Gába A, Dygrýn J, Rubín L, Šimůnek A, Sigmund E. Is adherence to the 24-hour movement guidelines associated with a reduced risk of adiposity among children and adolescents? BMC Public Health. 2020;20(1):1119. 32677940 10.1186/s12889-020-09213-3 PMC7364474

[17] Roman-Viñas B, Chaput JP, Katzmarzyk PT, Fogelholm M, Lambert EV, Maher C, Maia J, Olds T, Onywera V, Sarmiento OL, et al. Proportion of children meeting recommendations for 24-hour movement guidelines and associations with adiposity in a 12-country study. Int J Behav Nutr Phys Act. 2016;13(1):123. 27887654 10.1186/s12966-016-0449-8 PMC5123420

[18] Marques A, Ramirez-Campillo R, Gouveia ÉR, Ferrari G, Tesler R, Marconcin P, Loureiro V, Peralta M, Sarmento H. 24-h Movement Guidelines and Overweight and Obesity Indicators in Toddlers, Children and Adolescents: A Systematic Review and Meta-Analysis. Sports Med Open. 2023;9(1):30. 37184735 10.1186/s40798-023-00569-5 PMC10185721

[19] Huang S, Huang Y, Gu Y, Chen H, Lv R, Wu S, Song P, Zhao D, Hu L, Yuan C. Adherence to 24-Hour Movement Guidelines in Relation to the Risk of Overweight and Obesity Among Children and Adolescents. J Adolesc Health. 2023;73(5):887–95. 37565981 10.1016/j.jadohealth.2023.06.009

[20] Yang Y, Yuan S, Liu Q, Li F, Dong Y, Dong B, et al. Meeting 24-Hour Movement and Dietary Guidelines: Prevalence, Correlates and Association with Weight Status among Children and Adolescents: A National Cross-Sectional Study in China. Nutrients. 2022;14(14):2822. 10.3390/nu14142822 PMC9317649 35889779

[21] Carson V, Chaput JP, Janssen I, Tremblay MS. Health associations with meeting new 24-hour movement guidelines for Canadian children and youth. Prev Med. 2017;95:7–13. 27923668 10.1016/j.ypmed.2016.12.005

[22] Chemtob K, Reid RER, Guimarães RF, Henderson M, Mathieu ME, Barnett TA, Tremblay A, Van Hulst A. Adherence to the 24-hour movement guidelines and adiposity in a cohort of at risk youth: A longitudinal analysis. Pediatr Obes. 2021;16(4):e12730. 32997442 10.1111/ijpo.12730

[23] Wang M, Zhong JM, Fang L, Wang H. Prevalence and associated factors of smoking in middle and high school students: a school-based cross-sectional study in Zhejiang Province, China. BMJ Open. 2016;6(1):e010379. 26769793 10.1136/bmjopen-2015-010379 PMC4735178

[24] Lin Y, Rankin R, Li SX, Li XY, Wang SJ, Lou WW, Gong QH. The association between weight loss behaviors and body weight perception in Chinese adolescents: 2007–2022. BMC Public Health. 2024;24(1):2535. 39294694 10.1186/s12889-024-20005-x PMC11409477

[25] NationalHealth Commission Medical Ethics Expert Committee Office, Chinese Hospital Association: Guidelines for the Construction of Ethics Review Committees for Clinical Research Involving Human Beings. (2023 ed.). 2023. https://www.hxkq.org/Html/News/Articles/15374.html. Accessed 24 Feb 2026.

[26] Eaton DK, Kann L, Kinchen S, Ross J, Hawkins J, Harris WA, Lowry R, McManus T, Chyen D, Shanklin S, et al. Youth risk behavior surveillance–United States, 2005. J Sch Health. 2006;76(7):353–72. 16918870 10.1111/j.1746-1561.2006.00127.x

[27] Lin Y, Huang JY, Rankin R, Lou WW, Li XY, Wang SJ, Tong F, Gong QH. Associations of suicidal behaviors with body weight and body weight perception in Chinese adolescents: 2007–2022. Soc Psychiatry Psychiatr Epidemiol. 2025;60(3):737–49. 39576329 10.1007/s00127-024-02794-z

[28] Wang M, Hu RY, Pan J, Wang H, Yu M, Xie KX, Gong WW. Awareness, current use of electronic cigarettes and associated smoking factors in Zhejiang Chinese adolescents. PLoS ONE. 2019;14(10):e0224033. 31634360 10.1371/journal.pone.0224033 PMC6802828

[29] Wang M, Zhong JM, Wang H, Zhao M, Gong WW, Pan J, et al. Breakfast Consumption and Its Associations with Health-Related Behaviors among School-Aged Adolescents: A Cross-Sectional Study in Zhejiang Province, China. Int J Environ Res Public Health. 2016;13(8):761. 10.3390/ijerph13080761 PMC4997447 27472357

[30] National Health and Family Planning Commission of the People’s Republic of China. Screening for overweight and obesity among school-age children and adolescents. WS/T 586–2018. Beijing: China Standards Press; 2018.

[31] Tremblay MS, Carson V, Chaput JP, Connor Gorber S, Dinh T, Duggan M, Faulkner G, Gray CE, Gruber R, Janson K, et al. Canadian 24-Hour Movement Guidelines for Children and Youth: An Integration of Physical Activity, Sedentary Behaviour, and Sleep. Appl Physiol Nutr Metab. 2016;41(6 Suppl 3):S311–327. 27306437 10.1139/apnm-2016-0151

[32] Zhang YT, Ma SX, Chen C, Liu SJ, Zhang CF, Cao ZB, Jiang F. Chinese Guidelines on Physical Activity for Children and Adolescents China. J Evidence-Based Pediatr. 2017;12(6):401–9.

[33] Witkiewitz K, Heather N, Falk DE, Litten RZ, Hasin DS, Kranzler HR, Mann KF, O’Malley SS, Anton RF. World Health Organization risk drinking level reductions are associated with improved functioning and are sustained among patients with mild, moderate and severe alcohol dependence in clinical trials in the United States and United Kingdom. Addiction. 2020;115(9):1668–80. 32056311 10.1111/add.15011 PMC7841874

[34] WHO Guidelines Approved by the Guidelines Review Committee. In. Guideline: Sugars Intake for Adults and Children. edn. Geneva: World Health Organization; 2015. 25905159

[35] Chinese Nutrition Society: The Chinese dietary guidelines. (2022). 2022.

[36] Zhang X, Liu J, Ni Y, Yi C, Fang Y, Ning Q, Shen B, Zhang K, Liu Y, Yang L, et al. Global Prevalence of Overweight and Obesity in Children and Adolescents: A Systematic Review and Meta-Analysis. JAMA Pediatr. 2024;178(8):800–13. 38856986 10.1001/jamapediatrics.2024.1576 PMC11165417

[37] Roberts KC, Yao X, Carson V, Chaput JP, Janssen I, Tremblay MS. Meeting the Canadian 24-Hour Movement Guidelines for Children and Youth. Health Rep. 2017;28(10):3–7. 29044440

[38] Du S, Lu B, Zhai F, Popkin BM. A new stage of the nutrition transition in China. Public Health Nutr. 2002;5(1a):169–74. 12027281 10.1079/PHN2001290

[39] Guo MM, Koh KT, Wang XZ. The effects of COVID-19 on the Physical Activity and Recreational Screen Time among Chinese children and adolescents. J Exerc Sci Fit. 2024;22(4):288–96. 38706950 10.1016/j.jesf.2024.04.002 PMC11066678

[40] Wang G, Ren F, Liu Z, Xu G, Jiang F, Skora E, Lewin DS. Sleep Patterns and Academic Performance During Preparation for College Entrance Exam in Chinese Adolescents. J Sch Health. 2016;86(4):298–306. 26930242 10.1111/josh.12379

[41] Zhou T, Cheng G, Wu X, Li R, Li C, Tian G, et al. The Associations between Sleep Duration, Academic Pressure, and Depressive Symptoms among Chinese Adolescents: Results from China Family Panel Studies. Int J Environ Res Public Health. 2021;18(11):6134. 10.3390/ijerph18116134 PMC8201038 34204145

[42] Chaput JP, Dutil C. Lack of sleep as a contributor to obesity in adolescents: impacts on eating and activity behaviors. Int J Behav Nutr Phys Act. 2016;13(1):103. 27669980 10.1186/s12966-016-0428-0 PMC5037605

[43] World Health Organization: Physical inactivity a leading cause of disease and disability, warns WHO. 2002. https://www.who.int/news/item/04-04-2002-physical-inactivity-a-leading-cause-of-427disease-and-disability-warns-who. Accessed 1 June 2025. 12365404

[44] López-Gil JF, Tapia-Serrano MA, Sevil-Serrano J, Sánchez-Miguel PA, García-Hermoso A. Are 24-hour movement recommendations associated with obesity-related indicators in the young population? A meta-analysis. Obesity. 2023;31(11):2727–39. 37726964 10.1002/oby.23848

[45] Hjorth MF, Chaput JP, Damsgaard CT, Dalskov SM, Andersen R, Astrup A, Michaelsen KF, Tetens I, Ritz C, Sjödin A. Low physical activity level and short sleep duration are associated with an increased cardio-metabolic risk profile: a longitudinal study in 8–11 year old Danish children. PLoS ONE. 2014;9(8):e104677. 25102157 10.1371/journal.pone.0104677 PMC4125285

[46] Zhou L, Liang W, He Y, Duan Y, Rhodes RE, Liu H, et al. Relationship of 24-Hour Movement Behaviors with Weight Status and Body Composition in Chinese Primary School Children: A Cross-Sectional Study. Int J Environ Res Public Health. 2022;19(14):8586. 10.3390/ijerph19148586 PMC9319103 35886438

[47] Zhu X, Healy S, Haegele JA, Patterson F. Twenty-Four-Hour Movement Guidelines and Body Weight in Youth. J Pediatr. 2020;218:204–9. 31959469 10.1016/j.jpeds.2019.11.031 PMC7042069

[48] Westerterp KR. Changes in physical activity over the lifespan: impact on body composition and sarcopenic obesity. Obes Rev. 2018;19(Suppl 1):8–13. 30511504 10.1111/obr.12781

[49] Boyland EJ, Nolan S, Kelly B, Tudur-Smith C, Jones A, Halford JC, Robinson E. Advertising as a cue to consume: a systematic review and meta-analysis of the effects of acute exposure to unhealthy food and nonalcoholic beverage advertising on intake in children and adults. Am J Clin Nutr. 2016;103(2):519–33. 26791177 10.3945/ajcn.115.120022

[50] Tashjian SM, Mullins JL, Galván A. Bedtime Autonomy and Cellphone Use Influence Sleep Duration in Adolescents. J Adolesc Health. 2019;64(1):124–30. 30366713 10.1016/j.jadohealth.2018.07.018

[51] Cain N, Gradisar M. Electronic media use and sleep in school-aged children and adolescents: A review. Sleep Med. 2010;11(8):735–42. 20673649 10.1016/j.sleep.2010.02.006

[52] Hale L, Guan S. Screen time and sleep among school-aged children and adolescents: a systematic literature review. Sleep Med Rev. 2015;21:50–8. 25193149 10.1016/j.smrv.2014.07.007 PMC4437561

[53] Spiegel K, Tasali E, Penev P, Van Cauter E. Brief communication: Sleep curtailment in healthy young men is associated with decreased leptin levels, elevated ghrelin levels, and increased hunger and appetite. Ann Intern Med. 2004;141(11):846–50. 15583226 10.7326/0003-4819-141-11-200412070-00008

[54] Chen ST, Liu Y, Tremblay MS, Hong JT, Tang Y, Cao ZB, Zhuang J, Zhu Z, Wu X, Wang L, et al. Meeting 24-h movement guidelines: Prevalence, correlates, and the relationships with overweight and obesity among Chinese children and adolescents. J Sport Health Sci. 2021;10(3):349–59. 32679341 10.1016/j.jshs.2020.07.002 PMC8167320

[55] Wells JC. Sexual dimorphism of body composition. Best Pract Res Clin Endocrinol Metab. 2007;21(3):415–30. 17875489 10.1016/j.beem.2007.04.007

[56] Chen J, Jin L, Wang F, Huang K, Wu W, Chen R, Maimaiti M, Chen S, Cao B, Zhu M, et al. Risk factors for obesity and overweight in Chinese children: a nationwide survey. Obesity. 2022;30(9):1842–50. 35918882 10.1002/oby.23515 PMC9545785

[57] Ebbeling CB, Pawlak DB, Ludwig DS. Childhood obesity: public-health crisis, common sense cure. Lancet. 2002;360(9331):473–82. 12241736 10.1016/S0140-6736(02)09678-2

[58] Würbach A, Zellner K, Kromeyer-Hauschild K. Meal patterns among children and adolescents and their associations with weight status and parental characteristics. Public Health Nutr. 2009;12(8):1115–21. 19243677 10.1017/S1368980009004996

