# Supplementary material for: Adherence to 24-hour movement guidelines and its association with the risk of overweight and obesity in Chinese adolescents: 2007 to 2022
Source: BMC Public Health. 2026 Mar 11;26:1276. doi: 10.1186/s12889-026-26887-3 (PMC13094055; doi:10.1186/s12889-026-26887-3)
Supplement: Supplementary file 1 — Supplementary Material 1. [file 12889_2026_26887_MOESM1_ESM.docx]

| **Supplementary Table 1.** Sensitivity analysis of the association between MVPA and overweight and obesity in adolescents using different physical activity categorizations | | | |
| --- | --- | --- | --- |
| Categorization 1^a^ | | | |
| MVPA | **OR** | **95% CI** | **P** |
| 7 days/week | 1 |  |  |
| <7 days/week | 1.17 | 0.91, 1.50 | 0.233 |
| Categorization 2^b^ | | | |
| MVPA | **OR** | **95% CI** | **P** |
| >3 days/week | 1 |  |  |
| <=3 days/week | 1.18 | 0.98, 1.41 | 0.076 |
| MVPA: moderate-to-vigorous physical activity; OR: odds ratio; CI: confidence interval The binary GLM was used to investigate the associations ^a^ Categorization 1: MVPA was categorized into two groups based on whether participants engaged in MVPA on all 7 days per the week. ^b^ Categorization 2: MVPA was categorized into two groups based on whether participants engaged in MVPA on at least 3 days per week. The model was adjusted for age, sex, school type, ethnicity, single children status, parental education status, parental marriage status, living situation, consumption of SSB, junk food, and alcohol, and survey wave. | | | |
